# Supplementary material for: Complexome profiling of the Chlamydomonas psb28 mutant reveals TEF5 as an early PSII assembly factor
Source: Plant Cell. 2025 Mar 17;37(6):koaf055. doi: 10.1093/plcell/koaf055 (PMC12164590; doi:10.1093/plcell/koaf055)
Supplement: koaf055_Supplementary_Data [file koaf055_supplementary_data.zip › psb28_interactive.zip!/plots/intensity/qualityPlots/peptides/FAD7_Cre01.g038600.t1.2.html]

### Cre01.g038600.t1.2

Trivial name: FAD7  
SeqCoverage [median]: 24.20  
Mass: 47.63 kDa  
  
 

| Sequence | MissedCleavage | Mass [Da] | wtIntensity | muIntensity | Score |
| --- | --- | --- | --- | --- | --- |
| EPMKSPGPLPTHLVEPLIR | 1 | 2110.16 | 0.00 | 1367.54 | 40.84 |
| GEEWSYLR | 0 | 1038.48 | 0.00 | 808.03 | 76.77 |
| GGLTTIDRDYGIFNK | 1 | 1668.85 | 7001.33 | 31440.91 | 59.87 |
| KLYDHLEPMAR | 1 | 1371.70 | 998.40 | 0.00 | 40.59 |
| LYDHLEPMAR | 0 | 1243.60 | 1473.17 | 4819.88 | 70.06 |
| NADGTYDLSAPPPFTLQDLR | 0 | 2190.06 | 1720.25 | 0.00 | 43.70 |
| SFTNDHYVADSGDIVYYQK | 0 | 2221.00 | 0.00 | 7166.47 | 87.32 |
| SFTNDHYVADSGDIVYYQKDPNFPSVGK | 1 | 3162.46 | 1519.77 | 4555.01 | 58.35 |
| SPGPLPTHLVEPLIR | 0 | 1624.93 | 4248.35 | 14840.52 | 116.96 |
|  |  |  | 16961.3 | 64998.4 |  |
